# Reliability and Validity of the Arabic Version of the Game Experience Questionnaire: Pilot Questionnaire Study

**DOI:** 10.2196/42584

**Published:** 2023-03-20

**Authors:** Mahmoud Rebhi, Mohamed Ben Aissa, Amayra Tannoubi, Mouna Saidane, Noomen Guelmami, Luca Puce, Wen Chen, Nasr Chalghaf, Fairouz Azaiez, Makrem Zghibi, Nicola Luigi Bragazzi

**Affiliations:** 1 Department of Human Sciences, Higher Institute of Sport and Physical Education of Sfax, University of Sfax Sfax Tunisia; 2 Department of Human and Social Sciences Higher Institute of Sport and Physical Education of Kef University of Jendouba Jendouba Tunisia; 3 Postgraduate School of Public Health Department of Health Sciences (DISSAL) University of Genoa Genoa Italy; 4 Department of Neuroscience, Rehabilitation, Ophthalmology, Genetics, Maternal and Child Health (DINOGMI), University of Genoa Genoa Italy; 5 Department of Child Psychology, The Children's Hospital, Zhejiang University School of Medicine, National Clinical Research Center for Child Health, National Children's Regional Medical Center, Hangzhou Zhejiang China; 6 Higher Institute of Sport and Physical Education of Gafsa, University of Gafsa Gafsa Tunisia; 7 Laboratory for Industrial and Applied Mathematics Department of Mathematics and Statistics York University Toronto, ON Canada

**Keywords:** Arab countries, game experience, reliability, scale, validity

## Abstract

**Background:**

Nowadays, digital gaming occupies a central position in the entertainment industry where it has developed into a cherished kind of entertainment in markets all over the world. In addition, it provides other sectors with various social and economic benefits. The Game Experience Questionnaire (GEQ) is a free, quantitative, and comprehensive self-report measure that was developed to assess the player game experience. Despite having been widely used by many research projects in the past, it has not been adapted into Arabic. Furthermore, several components of the scale proved problematic from a psychometric point of view. Therefore, a modified version of the scale is needed to measure the gaming experience of the Arab population.

**Objective:**

The aim of this study was to validate and examine the psychometrics of an adapted Arabic version of the GEQ in Tunisia.

**Methods:**

A total of 771 volunteer participants completed an online survey, which included an Arabic version of the GEQ, gaming data, and a sociodemographic questionnaire. Subjects were randomized in order to complete two phases of the study: exploratory and confirmatory. The exploratory data were acquired from 360 respondents whose mean age was 23.89 (SD 2.29) years. Out of 360 respondents, 111 (30.8%) were female and 249 (69.2%) were male. Confirmatory data were obtained from the remaining 411 subjects whose mean age was 21.94 (SD 1.80) years. Out of 411 subjects, 169 (41.1%) were female and 242 (58.9%) were male.

**Results:**

After the elimination of two items, the exploratory and the confirmatory factor analyses provided an adequate factor structure of the Arabic version of the GEQ. In addition, the internal consistency coefficients suggested the reliability of the instrument. Significant differences were revealed for three subcomponents: flow by age (*η*^2^=0.013, *P*=.002), gender (*η*^2^=0.007, *P*=.02), and game type (*η*^2^=0.03, *P*<.001). For competence (*η*^2^=0.01, *P*=.03) and immersion (*η*^2^=0.02, *P*=.01), significant differences were highlighted by the type of game. The discriminant and convergent validities of the instrument were supported by calculating the average variance extracted (AVE) and comparing the square roots of the AVE values to the correlation coefficients, respectively.

**Conclusions:**

The Arabic adapted version of the GEQ is valid and reliable and can be administered to measure the game experience in Arab countries.

## Introduction

Nowadays, digital gaming occupies a central position in the entertainment industry, where it has developed into a cherished type of entertainment in markets all over the world. Recent studies showed that the number of players keeps increasing at a high rate; for instance, SteamDB, which is one of the biggest video game distribution platforms in the world, showed growth from 67 million monthly users in 2017 to 120 million in 2020 [1]. Moreover, a total of 2.7 billion players were recorded around the world by the end of 2020. This is an increase of more than 135 million players in comparison to the numbers reported in 2019 [2].

The impact that digital games have had on modern culture has resulted in their proliferation into many spheres of human existence, not only in the form of consumable media goods but also as cultures and in ways of living [3-5]. As a result of increased connectivity and processing power brought about by the proliferation of console, online, and mobile technologies, new types of games are emerging, such as advertising games, augmented reality games, and social media games [6-8]. These games have been developed for a multitude of different platforms, each via digital technologies that cater to different gaming needs and practices. These new types of games include, for example, social gaming, hardcore gaming, couch gaming, and e-sports [9-11].

New developments in technology and software go up against a yearning for the past that exists across player bases that span many generations and consist of people living wildly different lives [12,13]. A broad variety of different business models emerged so that companies could appeal to vastly diverse player bases. Each model alters the ways in which players may engage with digital games as individuals (eg, players, parents, and children) or collectives (eg, communities, networks, and subcultures), that is, the ways that players live their lives, the ways they think, and the ways they behave [14-16]. As emotions are a fundamental component of human behavior, it is possible that all types of games produce emotional experiences [17]. Thus, game experience is considered one of the most central targets in the development of any game [18].

Development of the game experience allows for the systematic and empirical evaluation of computer games, which helps in the conduct of player experience research by combining numerical recording of parameters (ie, physically from players and technically within entertainment software) and qualitative assessments of experience, including behavioral observations. It is possible to render a high-resolution image of the complex interactions driving gameplay and player experience [19-23].

Some self-report measures were developed and validated to assess the player game experience as “an ensemble made up of the player’s sensations, thoughts, feelings, actions, and meaning-making in a gameplay setting” [24,25].

Recent years have seen an increase in the awareness among game makers of the commercial worth of adapting games for the Arab audience [26]. One of the most vibrant and rapidly expanding gaming communities in the world can be found in the Middle East and North Africa [27].

To satisfy the requirements of the Arab gaming community and to broaden the markets available to game developers, a number of video game companies believe that it is essential to develop an evaluation tool in the Arabic language to evaluate the gaming experiences of players in countries where Arabic is the primary language.

Among the tools previously developed, the following instruments are multidimensional: the Game Engagement Questionnaire [28], the Temple Presence Inventory, and the Social Presence in Gaming Questionnaire [29]. However, no Arabic tool has been found to evaluate the game experience in countries where Arabic is the primary language.

The Core Elements of the Gaming Experience Questionnaire include the following two measurement scales [30]: the Motivations to Play in Online Games scale, which evaluates motivation as a trait [31], and the Game Experience Questionnaire (GEQ).

The Player Experience of Need Satisfaction (PENS) instrument [32] and the GEQ are two popular scales [33,34]. The PENS is based on self-determination theory [35], which defines how experiences meet universal requirements (ie, competence, autonomy, and relatedness). In contrast, the GEQ does not rely on any particular theory in its construction; instead, it is predicated on conceptual explanations of the player experience in addition to focus groups that were carried out with players of video games. In fact, assessment of player experience incorporates subjective psychological experiences and emotions. The aim is to examine a broad range of experience that encourages a person to desire to play a game, continue playing it, return to it, and promote it to others [36].

The GEQ is a multidimensional instrument that has been widely used; it was experimentally and conceptually anchored with high-quality questions. The instrument has been used extensively to examine a variety of playing styles in many research projects. The questionnaire is free to use, quantitative, and comprehensive. It was commonly employed to evaluate new games or playful systems.

The first edition of the GEQ consisted of 42 items that were split among seven different variables (ie, challenge, competence, flow, immersion, tension, positive affect, and negative affect). Subsequently, a 33-item version of the instrument was developed. However, the psychometrics of the GEQ were not tested and, therefore, lacked evidence of reliability and validity [36-38].

Moreover, in a systematic review that included 73 studies that used the GEQ, the factor structure of the instrument was criticized. Among the serious criticisms was the lack of psychometric verification; the items did not support a 7-factor structure and some factors were eliminated in the modified version [38].

In the same study and following the exploratory factor analysis (EFA), the overlapping of eight items was highlighted. Additionally, after the confirmatory factor analysis (CFA), the authors proposed a revised structure, in which negative affect, tension, and challenge reflected a single negativity factor; however, the three concepts cannot constitute a single factor. Indeed, challenge is a core element of gameplay in the majority of digital games [39] and is widely considered [38] to play a crucial role in the enjoyment of games [40]. In addition, tension includes feelings of annoyance, frustration, and pressure. On the other hand, negative affect summarizes feelings related to a bad mood and boredom, whereas positive affect includes feelings of happiness and enjoyment. In fact, some studies have discussed modifying the GEQ in some fashion, but the details of how and why these modifications were done were not given. Furthermore, modifying the GEQ in some manner has been referenced in several research studies, but the details of how and why the modifications were performed are rarely revealed.

Therefore, the purpose of this research is to validate an adapted Arabic version of the GEQ and assess the psychometric properties of the questionnaire.

## Methods

### Data Collection and Procedures

We collected cross-sectional data from an online survey. To distribute the questionnaire and reach the largest number of target population members, we used snowball sampling to collect information from Tunisian Facebook users. This strategy is increasingly used in research involving online recruitment. Invitations to complete an informed consent form sent from specified Gmail accounts were first posted in several Facebook groups. Following this, the respondents asked their friends to participate in the survey. Thereafter, we invited the subjects to register at the *Health games* website [41].

The creation of this environment allowed us to access users’ IP addresses, delete duplicates, and have a single response per user. However, for privacy and security reasons, no personal information was collected (eg, names, home addresses, and telephone numbers). In addition, this research adheres to the Checklist for Reporting Results of Internet E-Surveys [42].

Participants (N=771) spent an average of 72 (SD 15.2) minutes per week playing video games. However, they spent an average of 60.3 (SD 9.4) minutes per week playing their favorite video game. Participants’ favorite games spanned a wide variety of categories within the gaming community.

The largest proportion of subjects participated in action games (295/771, 38.3%), adventure games (175/771, 22.7%), online multiplayer games (119/771, 15.4%), and real-time strategy games (112/771, 14.5%), whereas the rest participated in other online games. Those recruited for the study were divided into two groups to conduct two exploratory and confirmatory studies and were asked to describe their experience of their favorite games:

1. Exploratory data were collected from 360 subjects whose mean age was 23.89 (SD 2.29) years and who were randomly selected from the participants. Both female (n=111, 30.8%) and male (n=249, 69.2%) subjects were recruited.
2. Confirmatory data were collected from 411 subjects (females: n=169, 41.1%; males: n=242, 58.9%) whose mean age was 21.94 (SD 1.80) years; subjects were divided into three grades and had different gaming experience.

### The Game Experience Questionnaire

Following the multiple criticisms from a psychometric point of view on the items designed for the GEQ, we used an adapted Arabic version of the instrument after reformulating the items of the instrument. A focus group was formed by two university researchers specializing in psychology, a bilingual translator, and a game expert to translate and modify the questionnaire.

The objective of this step was to make a translation of the GEQ, to develop an initial version in Arabic, and to reformulate the problematic items. Hence, the initial version was translated, revised, and submitted to a back translation. During this procedure, the translation met the methodological criteria of transcultural validation [43,44]. In parallel, problematic items were identified from the literature and reformulated. As a result, these items were deleted and replaced by new items referring to concepts in the literature. The procedure used in the modification and adaptation of the instrument is presented in the flowchart in Multimedia Appendix 1.

The changes concerned seven items of the original questionnaire. As an example, for immersion, the item “It was aesthetically pleasing” was replaced with the item “I found it fantastic.” For flow, we modified two items: “I was fully occupied with the game” and “I was deeply concentrated in the game” were replaced with “I don’t see the time passing” and “I’m not worried about other people’s opinions,” respectively. Multimedia Appendix 2 summarizes the list of modified items, and Multimedia Appendix 3 highlights the Arabic version of the GEQ.

The version developed in Arabic was then subjected to a pilot test on a group of university students in physical education and sports (N=27). Eventually, an English-language translation was developed and accepted by the formed committee.

Immersion, flow, competence, positive affect, negative affect, tension, and challenge are the seven aspects that are measured by the Arabic version of the GEQ, which is a 33-item scale that measures the experience of game players across these seven categories. The items in the questionnaire are presented in the form of statements, and respondents are asked to rate those statements in order to reflect their level of satisfaction with the game. Responses are rated on a 5-point Likert scale, with the following anchors: 0 (“not at all”), 1 (“somewhat”), 2 (“moderately”), 3 (“fairly”), and 4 (“very”).

### Ethics Statement

The Ethics Committee of the High Institute of Sports and Physical Education of Kef, University of Jendouba, Jendouba, Tunisia, approved this study (approval number: PHS-07/2022). The study was carried out in accordance with the legal norms of the Declaration of Helsinki and its revisions (2013).

### Statistical Analysis

Skewness and kurtosis tests were used to explore data normality, whereas multivariate normality was assessed in the confirmatory phase. Data with asymmetry and kurtosis values over 7 and 3, respectively [45], were considered non-Gaussian and indicated poor psychometric sensitivity. The Mardia coefficient was used to examine multivariate normality and to find substantial deviations [46].

Unweighted least squares with direct Oblimin rotation were used for GEQ exploratory analysis. Thus, the polychoric correlation matrix was analyzed to extract factors. The sample adequacy was assessed using the Kaiser-Meyer-Olkin (KMO) statistic. Hair et al [47] stated that the KMO value must be larger than 0.60 to accept the factorial solution. The chi-square value of the Bartlett sphericity test was also computed. The factors were kept for eigenvalues greater than 1 and the scree plot. Moreover, items with factor loadings less than 0.5 were eliminated [48].

We performed the CFA with the maximum likelihood method to establish model parameter estimation. CFA goodness of fit is evaluated using a range of model fit indices. Model assessment included chi-square, chi-square/*df*, goodness-of-fit index (GFI), adjusted GFI (AGFI), comparative fit index (CFI), Tucker-Lewis index (TLI), root mean square error of approximation (RMSEA), and standardized root mean square residual (SRMR). Because large samples impact the chi-square fit statistic, the ratio of the chi-square statistic to the *df* (ie, chi-square/*df*) is preferable. For GFI and AGFI, Hu and Bentler [49] recommend a critical value of 0.90 or higher to accept the model. CFI and TLI have a threshold value of 0.95 or higher. Moreover, SRMR less than 0.08 and RMSEA less than 0.08 suggest a reasonable fit.

The reliability of the instrument was examined by evaluating three internal consistency indices simultaneously—McDonald *ω*, Cronbach *α*, and Gutmann λ6—since the classical coefficient Cronbach *α*, which has been reported in the majority of studies [50], was criticized for developing multidimensional scales [51]. For the three indices, values of 0.90 and greater are an indicator of outstanding internal consistency, values of 0.80 to 0.90 are an indicator of good reliability, and values of 0.70 to 0.80 are acceptable values. Lower levels indicate that the internal consistency is unacceptable [52-54].

We used univariate variance analysis, with age as a covariate, with partial eta-squared as a magnitude of effect to compare subscale scores by game type and genre. According to Cohen [55], eta-squared values of less than 0.01 represent trivial effects, values between 0.01 and 0.06 represent medium effects, values between 0.06 and 0.14 represent large effects, and values that exceed 0.14 represent very large effects. For every significant difference revealed by the test, we performed a post hoc Bonferroni test.

Convergent validity was evaluated by calculating the average variance extracted (AVE). To confirm the convergent validity, AVE values should exceed 0.50 [56]. Discriminant validity was established by the Fornell-Larcker criterion [57]. This procedure consists of comparing the square roots of the AVE values with the correlation coefficients between latent constructs [56].

The relationship between instrument dimensions, game addiction, and mental health parameters was assessed by the Pearson correlation matrix. To examine these associations, we used low (<0.35), moderate (0.36-0.67), and strong (>0.67) thresholds for the correlation coefficients [58].

Statistical analyses of the GEQ scores were performed using free JASP software (version 0.16.3.0; The JASP Team) and the lavaan R package from RStudio (version 1.3.1093; RStudio, PBC).

Results with *P* values less than or equal to .05 were deemed statistically significant in all statistical analyses.

## Results

### Overview

The 33 items of the GEQ were submitted to unweighted least squares EFA with Kaiser normalization and the direct Oblimin rotation method. Sampling adequacy was supported by the KMO value, which was equal to 0.83, and the Bartlett test of sphericity was significant (*χ*^2^_528_=4673.8, *P*<.001).

The results of the factorial solution suggested the elimination of two items (items 2 and 22; see factor loadings in Table 1) and the extraction of seven factors that explained 61.6% of the total variance.

The first three components extracted from the EFA were negative affect (eigenvalue=5.08), immersion (eigenvalue=3.78), and positive affect (eigenvalue=3.55), which explained 15.4%, 11.5%, and 10.7% of the total variance, respectively. Competence (eigenvalue=2.56), flow (eigenvalue=2.1), and challenge (eigenvalue=1.79) explained 7.7%, 6.1%, and 5.4% of the total variance, respectively. The last component was tension (eigenvalue=1.57), which explained 4.8% of the total variance.

**Figure 1:**
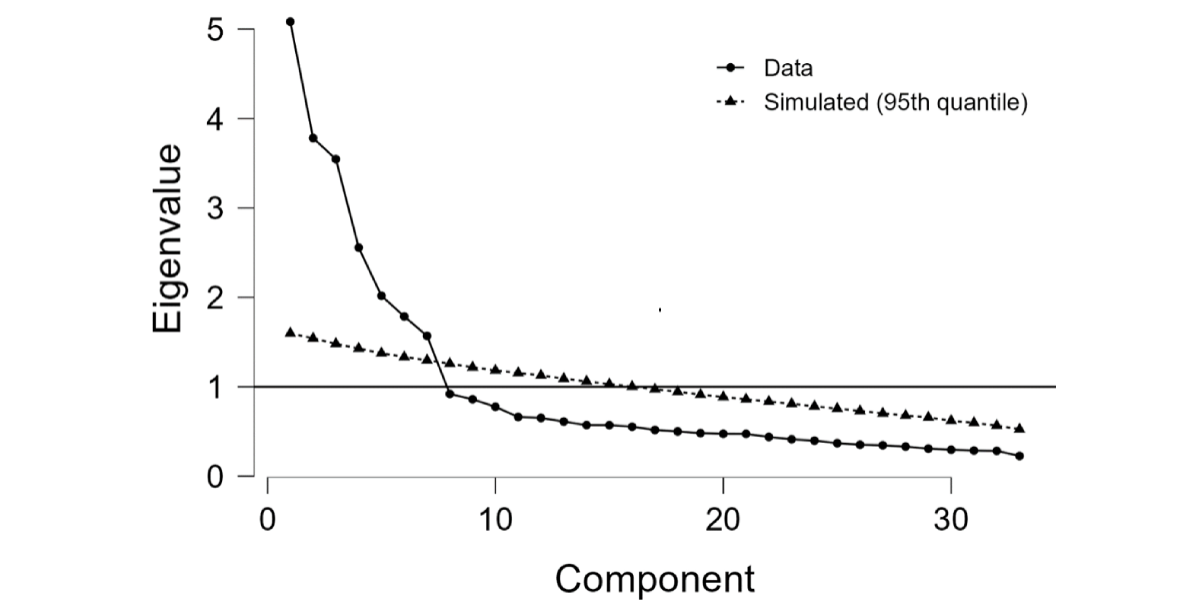
Scree plot of the Arabic version of the Game Experience Questionnaire.

As shown in Figure 1, the purpose of the cut function is to select factors with eigenvalues greater than 1. The collected data and the simulated data, which were generated by the JASP software, showed a 7-factor solution: the factors retained must be above the cutoff line perpendicular to the axis of the eigenvalues (intersection for eigenvalue=1).

**Table 1:** Descriptive statistics, normality assessment, and factor loadings (ie, lambda) of the exploratory sample analysis.

Item	Scores, mean (SD)	Skewness	Kurtosis	*λ*
1	2.97 (0.80)	–0.82	0.99	0.79
2	2.75 (1.04)	–0.83	0.32	<0.5
3	3.00 (0.83)	–0.59	–0.13	0.66
4	3.03 (0.79)	–0.90	1.42	0.77
5	3.04 (0.79)	–0.86	1.14	0.78
6	3.03 (0.84)	–0.77	0.53	0.76
7	2.97 (0.89)	–0.51	–0.51	0.69
8	2.93 (0.88)	–0.67	0.23	0.68
9	2.94 (0.91)	–0.65	–0.18	0.75
10	3.01 (0.87)	–0.84	0.49	0.76
11	2.78 (0.93)	–0.51	–0.36	0.79
12	2.87 (0.87)	–0.59	0.20	0.80
13	2.95 (0.83)	–0.60	–0.04	0.69
14	2.79 (0.91)	–0.35	–0.67	0.74
15	2.74 (0.94)	–0.40	–0.59	0.82
16	2.08 (0.91)	–0.71	–0.26	0.79
17	2.15 (0.89)	–0.88	0.05	0.81
18	2.19 (0.90)	–1.03	0.33	0.82
19	2.00 (0.88)	–0.70	–0.13	0.82
20	2.16 (0.91)	–0.84	–0.20	0.85
21	2.74 (0.93)	–0.46	–0.48	0.79
22	2.44 (1.17)	–0.52	–0.61	<0.5
23	2.68 (1.07)	–0.43	–0.86	0.81
24	2.69 (0.95)	–0.44	–0.62	0.79
25	2.63 (1.00)	–0.44	–0.39	0.74
26	2.49 (1.11)	–0.41	–0.77	0.75
27	2.23 (0.74)	–1.06	1.52	0.85
28	2.24 (0.75)	–1.07	1.43	0.78
29	2.21 (0.81)	–0.69	1.93	0.76
30	2.86 (0.87)	–0.48	–0.14	0.81
31	2.78 (0.87)	–0.32	–0.32	0.81
32	2.76 (0.89)	–0.53	0.09	0.77
33	2.84 (0.84)	–0.31	–0.53	0.75

### Internal Consistency

The consistency coefficient of the scale was high overall, which is a positive sign. For all subscales, McDonald *ω*, Cronbach *α*, and Guttman λ6 varied from acceptable to good (Table 2).

The highest values were demonstrated for negative affect (McDonald *ω*=0.89, Cronbach *α*=.89, and Guttman λ6=0.87). The smallest values were demonstrated for tension (McDonald *ω*=0.77, Cronbach *α*=.77, and Guttman λ6=0.70).

The average interitem correlations of the seven subscales ranged from 0.45 to 0.61 and confirmed the reliability of the GEQ instrument.

**Table 2:** Internal consistency of the Game Experience Questionnaire.

Aspect	McDonald *ω*	Cronbach *α*	Guttman λ6	Average interitem correlation
Flow	0.80	.79	0.75	0.49
Competence	0.81	.80	0.78	0.45
Positive affect	0.86	.85	0.83	0.54
Negative affect	0.89	.89	0.87	0.61
Immersion	0.85	.85	0.82	0.53
Tension	0.77	.77	0.70	0.53
Challenge	0.80	.80	0.75	0.50

### Confirmatory Factor Analysis

Before performing the CFA, our cross-sectional data were submitted for univariate and multivariate normality analysis. The item scores and the skewness and kurtosis normality coefficients are displayed in Table 3. In addition, we calculated the Mardia kurtosis and skewness for multivariate normality [59]. The skewness standardized coefficient *β*-hat was equal to 104.94 (*P*<.001), whereas the κ concentration parameter was equal to 1147.85 (*P*<.001). Since both *P* values were less than .05, the items did not follow a multivariate normal distribution and were considered ordinal.

**Figure 2:**
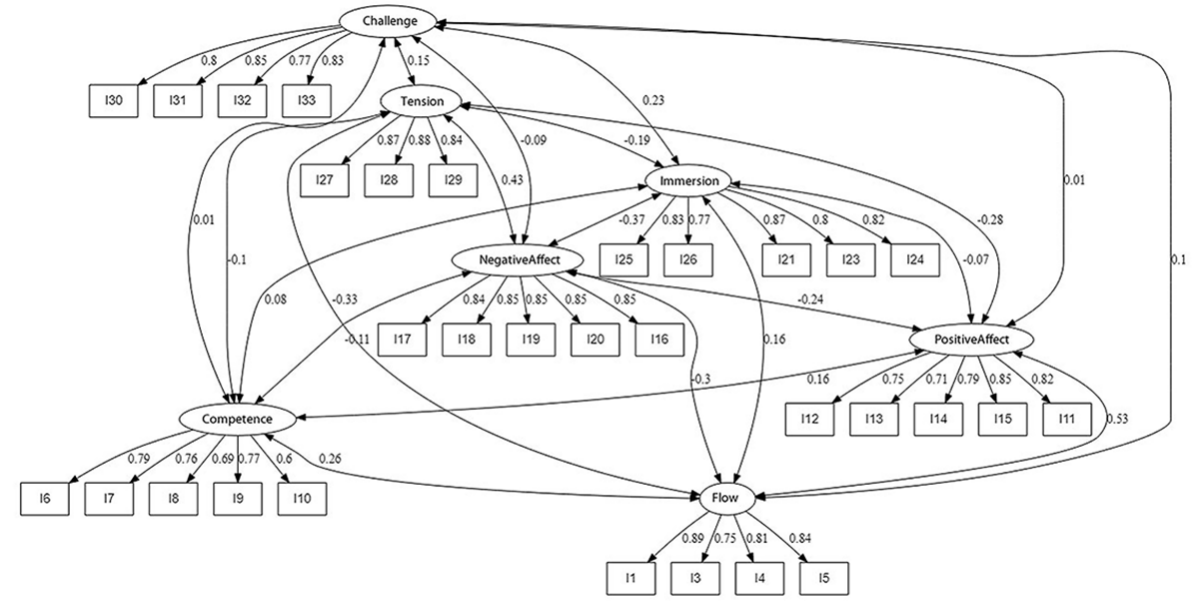
The final confirmatory factor analysis of the Arabic Game Experience Questionnaire. Factor loadings ranged from 0.60 to 0.89. I: item.

The CFA gave a first-order model with a nonsignificant chi-square value (*χ*^2^_413_=454.3; *χ*^2^/*df*=1.1; *P*=.08) with adequate error: the RMSEA was 0.016 (90% CI 0-0.024) and the SRMR was 0.031 (Figure 2). Moreover, the AGFI value was 0.92 and the GFI value was 0.93. Finally, the CFI and TLI values were 0.995 and 0.994, respectively. These results showed that a first-order model fit the data well.

**Table 3:** Descriptive statistics and normality assessment of the confirmatory sample (n=411).

Item	GEQ^a^ score, mean (SD)	Skewness	Kurtosis
1	2.69 (0.98)	–0.73	0.19
2	2.76 (1.00)	–0.80	0.35
3	2.78 (0.99)	–0.77	0.26
4	2.75 (0.97)	–0.60	–0.04
5	2.81 (1.02)	–0.87	0.38
6	2.90 (0.96)	–0.68	–0.18
7	2.93 (0.91)	–0.57	–0.24
8	2.90 (0.93)	–0.61	–0.08
9	2.86 (0.94)	–0.49	–0.41
10	2.90 (0.88)	–0.59	–0.16
11	2.86 (0.93)	–0.57	–0.29
12	2.90 (0.94)	–0.76	0.27
13	2.85 (0.90)	–0.59	–0.03
14	2.90 (0.98)	–0.65	–0.27
15	2.81 (0.98)	–0.61	–0.25
16	1.92 (1.06)	–0.31	–0.78
17	1.91 (1.04)	–0.38	–0.70
18	1.99 (1.04)	–0.55	–0.63
19	1.87 (1.02)	–0.33	–0.70
20	1.93 (1.04)	–0.39	–0.79
21	2.71 (1.11)	–0.57	–0.58
22	2.75 (1.04)	–0.57	–0.26
23	2.70 (1.11)	–0.44	–0.89
24	2.75 (1.05)	–0.45	–0.77
25	2.64 (1.05)	–0.60	–0.22
26	2.57 (1.11)	–0.37	–0.75
27	1.94 (1.09)	–0.15	–0.74
28	2.00 (1.13)	–0.21	–0.66
29	1.96 (1.11)	–0.28	–0.83
30	2.68 (0.99)	–0.59	0.02
31	2.76 (1.03)	–0.47	–0.63
32	2.65 (1.00)	–0.44	–0.51
33	2.69 (1.03)	–0.53	0.13

^a^GEQ: Game Experience Questionnaire.

### Sensitivity of the Scale

Descriptive statistics of the Arabic GEQ subscales by gender and type of game are presented in Multimedia Appendix 4. Significant differences were revealed for flow by age (*η*^2^=0.013, *P*=.002), gender (*η*^2^=0.02, *P*<.001), and game type (*η*^2^=0.03, *P*<.001), with no interaction effect. Significant differences were highlighted by the type of game for competence (*η*^2^=0.01, *P=*.03) and immersion (*η*^2^=0.02, *P*=.01).

Concerning flow, the Bonferroni test, which compares scores according to the type of game, revealed a difference between action games and other games (*P*=.05). Similarly, for immersion, significant differences were demonstrated between adventure games and strategy games (*P*=.03) and between adventure games and other games (*P*=.02; Table 4).

**Table 4:** Summary of *F* tests calculated using type III sum of squares for Arabic Game Experience Questionnaire subscales.

Aspect		Age (*df*=1)^a^	*P* value	Gender (*df*=1)	*P* value	Type of game (*df*=4)	*P* value	Gender × type of game (*df*=4)	*P* value
**Flow**									
	*F* test	10.15	.002	5.41	.02	5.78	<.001	1.02	.40
	Partial *η*^2^	0.013	—	0.007	—	0.030	—	0.005	—
**Competence**									
	*F* test	1.14	.29	0.33	.57	2.75	.03	0.40	.81
	Partial *η*^2^	0.00	—	0.00	—	0.01	—	0.002	—
**Positive affect**									
	*F* test	0.048	.83	3.17	.08	1.071	.37	0.79	.53
	Partial *η*^2^	0.000	—	0.004	—	0.006	—	0.004	—
**Negative affect**									
	*F* test	0.13	.72	0.33	.57	0.09	.99	0.70	.60
	Partial *η*^2^	0.000	—	0.000	—	0.000	—	0.004	—
**Immersion**									
	*F* test	0.83	.36	0.64	.42	3.25	.01	2.04	.09
	Partial *η*^2^	0.001	—	0.001	—	0.017	—	0.011	—
**Tension**									
	*F* test	0.430	.51	0.23	.64	0.58	.66	3.05	.02
	Partial *η*^2^	0.001	—	0.000	—	0.003	—	0.016	—
**Challenge**									
	*F* test	0.497	.48	0.54	.46	1.02	.40	0.27	.90
	Partial *η*^2^	0.001	—	0.001	—	0.005	—	0.001	—

^a^Error *df*=760; total *df*=771.

### Discriminant and Convergent Validity

The AVE values were all greater than 0.50. For flow and competence, the values were 0.68 and 0.53, respectively. For positive affect, the value was 0.62, and for negative affect, the value was 0.72. Finally, for immersion, tension, and challenge, the values were 0.71, 0.66, and 0.75, respectively.

The discriminant validity of the instrument was confirmed by the values of the square roots of the AVEs, which are presented diagonally in Table 5. All of these values were greater than the values of the correlations between the dimensions. According to these criteria, items should share a greater amount of variance with their intended underlying construct than with the other constructs. The shared variances between factors confirmed the discriminant validity.

**Table 5:** Discriminant validity of the Arabic Game Experience Questionnaire and correlation analysis (Pearson *r* and 2-tailed *P* value).

Aspect		Flow	Competence	Positive affect	Negative affect	Immersion	Tension	Challenge
**Flow**								
	*r*	0.82^a^	0.27^b^	0.45^b^	–0.18^b^	0.05	0.20^b^	0.07
	*P* value	—^c^	<.001	<.001	<.001	.34	<.001	.16
**Competence**								
	*r*	0.27^b^	0.73^a^	0.23^b^	–0.09	0.02	–0.08	0.02
	*P* value	<.001	—	<.001	.08	.70	.10	.72
**Positive affect**								
	*r*	0.45^b^	0.23^b^	0.79^a^	–0.89	0.08	–0.19^b^	0.05
	*P* value	<.001	<.001	—	.07	.88	<.001	.32
**Negative affect**								
	*r*	–0.18^b^	–0.09	–0.09	0.85^a^	–0.17^b^	0.35^b^	–0.01
	*P* value	<.001	.08	.07	—	<.001	<.001	.87
**Immersion**								
	*r*	0.05	0.02	0.01	–0.17^b^	0.84^a^	0.01	0.02
	*P* value	.34	.70	.88	<.001	—	.90	.70
**Tension**								
	*r*	–0.20^b^	–0.08	–0.19^b^	0.35^b^	0.01	0.81^a^	0.08
	*P* value	<.001	.10	<.001	<.001	.90	—	.13
**Challenge**								
	*r*	0.07	0.02	0.05	–0.01	0.02	0.08	0.86^a^
	*P* value	.16	.72	.32	.87	.70	.13	—

^a^Square root of the average variance extracted.

^b^The correlation is significant at a significance level of *P*<.001.

^c^Not applicable.

## Discussion

### Principal Findings

The objective of this study was to evaluate the psychometric properties of an adapted Arabic-language version of the GEQ. The results of the principal factor analysis suggest the elimination of one item from the flow subscale and another item from the immersion subscale. Examination of the reliability by means of the three internal consistency coefficients confirmed the factorial solution of seven components and retained 31 items. Similarly, examination of the first-order model by means of CFA supported the structure of our adapted version.

Significant differences were revealed for flow by age, gender, and game type. For competence and immersion, significant differences were highlighted by the type of game and partially by the sensitivity of our tool.

Finally, the construct validity of the tool was established by convergent and discriminant validity.

In line with our results, the EFA, using Oblimin rotation, extracted seven factors explaining 62% of the total variance. However, the CFA and EFA results implied that the factor structure of the GEQ was inadequate and that many items will need to be dropped. This is in line with other studies [38]. In addition, the study showed that the challenge and negative affect components did not have adequate internal consistency. In addition, results from the CFA in this study suggested that the proposed items did not present an adequate 7-factor model. The CFA results suggested that the proposed model did not acceptably fit the data (*χ*^2^_443_=1582.0, *P*<.001; *χ*^2^/*df*=3.57; CFI=0.88; RMSEA=0.068). Law et al [38] concluded that the positive affect and immersion subscales were reliable; however, they suggested modifications to two flow items.

In convergence with these results, the EFA of the original scale showed various problematic items and suggested modification of the scale factors [37]. After the removal of several items and the fusion of subscales, they found that the CFA of the GEQ revealed poor model fit (RMSEA=0.062; CFI=0.834). However, the revised version of the GEQ with covariances showed an acceptable fit index (Multimedia Appendix 5). However, this work did not explain why they inferred the negative affect and tension subscales, as they are two separate concepts [60,61].

In fact, no study that examined the psychometrics has been interested in verifying the reliability of the original version of the instrument. Regarding this point, research suggested examining the reliability of measuring instruments. For measurement scales, reliability is generally calculated by internal consistency indices, test-retest reliability, or interrater reliability [62-65].

IJsselsteijn et al [33] included negative affect and tension in their GEQ. In contrast, recent work [66] has excluded negative features from the Gameful Experience Scale (GAMEX). In the GAMEX and in game research, these negative elements were described as emotional responses. However, Sabet et al [67] showed that the GEQ has a “forgiving effect” and that players can forgive or forget a bad experience if it coincides with a long duration of a pleasant experience. This demonstrated that negative affect could be present but camouflaged by the positive aspects of the game.

Our results revealed differences depending on the type of game. In parallel, Engl and Nacke [68] found significant interactions for gender, player type, and age in mobile gaming experience. Moreover, Quax et al [69] revealed differences in the experience of gamers according to four categories of games—action games, puzzle games, strategy games, and racing games—in terms of pleasure and frustration.

Our findings did not demonstrate any differences between single and multiplayer games. However, several game experience studies indicated that playing games against other people was more fun and more exciting than playing alone [70,71]. In line with our results, flow, immersion, and positive affect were often used by researchers as indicators of fun and enjoyment during gameplay [72]. In fact, Gajadhar et al [71] concluded that player experience measured by the GEQ is different for positive affect, skill, and tension between multiplayer gaming and gaming against the computer.

Few studies have examined whether gender and age affect gaming experience. Recent studies revealed that male players and younger players performed better in games [73]. As an example, using social theories, Chappetta and Barth [74] showed differences in how games were played and experienced by women compared to men.

However, due to the wide variety of games available, it was legitimate to assert that playing games did not provide a singular experience. Instead, a game might only be identified as useful once the player has participated in a variety of distinct situations, which highlights the fact that the gaming experience encompassed several dimensions. The impact that digital games have had on modern culture has resulted in their proliferation into many spheres of human existence.

### Limitations of the Study

This study used online questionnaires, which have general and specific limitations. The sampling approach was nonprobability since study subjects were limited to Facebook users. The nonprobability nature of the survey was emphasized by the open invitation to participants. Participant self-selection made it difficult to identify nonresponse issues and generated an unrepresentative sample. To obtain generalizable results, a study must use probability sampling, a high-quality sampling frame, and enough follow-ups to improve response rates. Such research, in partnership with a service developer willing to provide a survey base of current and former users, would be preferred. Also, concerning the modifications made to the GEQ, the inverted items were absent in the instrument, which could be considered an additional limitation.

It should be noted that the GEQ manual suggested administering the GEQ soon after the conclusion of a game session. However, the duration between the end of the game and the administration of the questionnaire has not been verified. Also, the instrument has only been only validated on amateur players; professional players have not been taken into consideration.

Another limitation of the study concerns the sensitivity of the instrument, which was not examined. For further research, this scale should be administered in several Arab countries. In addition, the questionnaire must be tested on a larger sample of players and with a wider range of games.

### Conclusions

EFA and CFA supported the GEQ’s structure. Internal consistency indicated the instrument’s reliability. However, the scale seems partially sensitive by game type. Convergent and discriminant validity indicated the tool’s construct validity. The instrument appeared to be a valid and reliable tool for assessing game experience in Arab countries.

## Appendix

Multimedia Appendix 1 Flow diagram of the Game Experience Questionnaire (GEQ) modification and translation.

Multimedia Appendix 2 Modified items of the Game Experience Questionnaire (GEQ).

Multimedia Appendix 3 Arabic modified version of the Game Experience Questionnaire (GEQ).

Multimedia Appendix 4 Descriptive statistics for Squares for the Arabic Game Experience Questionnaire (GEQ) Sub-scales by game type and Gender.

Multimedia Appendix 5 Comparison fit index of the Arabic Game Experience Questionnaire (GEQ) and the original version.

## Abbreviations

AGFI: adjusted goodness-of-fit index
AVE: average variance extracted
CFA: confirmatory factor analysis
CFI: comparative fit index
EFA: exploratory factor analysis
GAMEX: Gameful Experience Scale
GEQ: Game Experience Questionnaire
GFI: goodness-of-fit index
KMO: Kaiser-Meyer-Olkin
PENS: Player Experience of Need Satisfaction
RMSEA: root mean square error of approximation
SRMR: standardized root mean square residual
TLI: Tucker-Lewis index

## References

[1] SteamDB.

[2] Chen Y, Hsu C (2020). Self-regulated mobile game-based English learning in a virtual reality environment. Comput Educ.

[3] Kreissl J, Possler D, Klimmt C (2021). Engagement with the gurus of gaming culture: Parasocial relationships to let’s players. Games Cult.

[4] Rahmatullah AS, Mulyasa E, Syahrani S, Pongpalilu F, Putri RE (2022). Digital era 4.0. LingCuRe.

[5] Barr M, Copeland-Stewart A (2021). Playing video games during the COVID-19 pandemic and effects on players’ well-being. Games Cult.

[6] Cheah I, Shimul AS, Phau I (2021). Motivations of playing digital games: A review and research agenda. Psychol Mark.

[7] Peñaherrera-Pulla OS, Baena C, Fortes S, Baena E, Barco R (2021). Measuring key quality indicators in cloud gaming: Framework and assessment over wireless networks. Sensors (Basel).

[8] Stewart NK, Smith R (2022). Networked students gaming together: Mobile scavenger hunts for online classrooms. Commun Teach.

[9] Jaramillo Álvarez MJ (2021). The Community Is the Game: A Corporate Communication Proposal [doctoral thesis].

[10] Richardson I, Hjorth L, Davies H (2021). Understanding Games and Game Cultures.

[11] Snodgrass JG, Dengah HJF, Upadhyay C, Else RJ, Polzer E (2021). Indian gaming zones as oppositional subculture. Curr Anthropol.

[12] Ponce J (2022). An Endless Ladder: The Preservation of Digital Interactive Artworks [doctoral thesis].

[13] Stringfield J (2022). Get in the Game: How to Level Up Your Business With Gaming, Esports, and Emerging Technologies.

[14] Wang Q, Ren H, Long J, Liu Y, Liu T (2019). Research progress and debates on gaming disorder. Gen Psychiatr.

[15] Wang Q, Liu L, Chen X (2019). Evolutionary dynamics of cooperation in the public goods game with individual disguise and peer punishment. Dyn Games Appl.

[16] Badrinarayanan VA, Sierra JJ, Martin KM (2015). A dual identification framework of online multiplayer video games: The case of massively multiplayer online role playing games (MMORPGs). J Bus Res.

[17] Ermi L, Mäyrä F (2005). Players' emotional experiences with digital games. Proceedings of the 6th DAC Conference - Digital Experience: Design, Aesthetics, Practice.

[18] Örtqvist D, Liljedahl M (2010). Immersion and gameplay experience: A contingency framework. Int J Comput Games Technol.

[19] Fernández Galeote D, Rajanen M, Rajanen D, Legaki N, Langley DJ, Hamari J (2021). Gamification for climate change engagement: Review of corpus and future agenda. Environ Res Lett.

[20] Oagaz H, Schoun B, Choi M (2022). Performance improvement and skill transfer in table tennis through training in virtual reality. IEEE Trans Visual Comput Graphics.

[21] Park E, Lee S, Ham A, Choi M, Kim S, Lee B (2021). Secrets of Gosu: Understanding physical combat skills of professional players in first-person shooters. Proceedings of the 2021 CHI Conference on Human Factors in Computing Systems.

[22] Schättin A, Häfliger S, Meyer A, Früh B, Böckler S, Hungerbühler Y, de Bruin ED, Frese S, Steinlin Egli R, Götz U, Bauer R, Martin-Niedecken AL (2021). Design and evaluation of user-centered exergames for patients with multiple sclerosis: Multilevel usability and feasibility studies. JMIR Serious Games.

[23] Tao G, Garrett B, Taverner T, Cordingley E, Sun C (2021). Immersive virtual reality health games: A narrative review of game design. J Neuroeng Rehabil.

[24] Bontchev B, Antonova A, Terzieva V, Dankov Y (2021). “Let Us Save Venice”—An educational online maze game for climate resilience. Sustainability.

[25] Vidergor HE (2021). Effects of digital escape room on gameful experience, collaboration, and motivation of elementary school students. Comput Educ.

[26] Al-Batineh M, Alawneh R (2022). Current trends in localizing video games into Arabic: Localization levels and gamers’ preferences. Perspectives.

[27] Arezki R, Belhaj F, Shah P (2019). Promoting a New Economy for the Middle East and North Africa.

[28] Brockmyer JH, Fox CM, Curtiss KA, McBroom E, Burkhart KM, Pidruzny JN (2009). The development of the Game Engagement Questionnaire: A measure of engagement in video game-playing. J Exp Soc Psychol.

[29] de Kort YAW, IJsselsteijn WA, Poels K (2007). Digital games as social presence technology: Development of the social presence in gaming questionnaire (SPGQ). Proceedings of the 10th Annual International Workshop on Presence.

[30] Calvillo-Gámez E, Cairns P, Cox A, Bernhaupt R (2015). Assessing the core elements of the gaming experience. Game User Experience Evaluation.

[31] Yee N (2006). Motivations for play in online games. Cyberpsychol Behav.

[32] Ryan RM, Rigby CS, Przybylski A (2006). The motivational pull of video games: A self-determination theory approach. Motiv Emot.

[33] IJsselsteijn W, van den Hoogen W, Klimmt C, de Kort Y, Lindley C, Mathiak K, Poels K, Ravaja N, Turpeinen M, Vorderer P (2008). Measuring the experience of digital game enjoyment. Proceedings of the 6th International Conference on Methods and Techniques in Behavioral Research.

[34] IJsselsteijn WA, de Kort YAW, Poels K (2013). The Game Experience Questionnaire.

[35] Ryan RM, Deci EL (2000). Self-determination theory and the facilitation of intrinsic motivation, social development, and well-being. Am Psychol.

[36] Norman KL (2013). GEQ (Game Engagement/Experience Questionnaire): A review of two papers. Interact Comput.

[37] Johnson D, Gardner MJ, Perry R (2018). Validation of two game experience scales: The Player Experience of Need Satisfaction (PENS) and Game Experience Questionnaire (GEQ). Int J Hum Comput Stud.

[38] Law ELC, Brühlmann F, Mekler ED (2018). Systematic review and validation of the Game Experience Questionnaire (GEQ) - Implications for citation and reporting practice. Proceedings of the 2018 Annual Symposium on Computer-Human Interaction in Play.

[39] Feil JH, Scattergood M (2005). Beginning Game Level Design.

[40] Vorderer P, Hartmann T, Klimmt C (2003). Explaining the enjoyment of playing video games: The role of competition. Proceedings of the 2nd International Conference on Entertainment Computing.

[41] Health games.

[42] Eysenbach G (2004). Improving the quality of web surveys: The Checklist for Reporting Results of Internet E-Surveys (CHERRIES). J Med Internet Res.

[43] Epstein J, Santo RM, Guillemin F (2015). A review of guidelines for cross-cultural adaptation of questionnaires could not bring out a consensus. J Clin Epidemiol.

[44] Galanis P (2019). Translation and cross-cultural adaptation methodology for questionnaires in languages other than Greek. Arch Hell Med.

[45] Hancock G, Mueller R (2013). Structural Equation Modeling: A Second Course.

[46] Mardia KV (1974). Applications of some measures of multivariate skewness and kurtosis in testing normality and robustness studies. Indian J Stat.

[47] Hair Jr JF, Sarstedt M, Hopkins L, Kuppelwieser VG (2014). Partial least squares structural equation modeling (PLS-SEM): An emerging tool in business research. Eur Bus Rev.

[48] Ware JE, Gandek B (1998). Methods for testing data quality, scaling assumptions, and reliability. J Clin Epidemiol.

[49] Hu L, Bentler PM (1999). Cutoff criteria for fit indexes in covariance structure analysis: Conventional criteria versus new alternatives. Struct Equ Modeling.

[50] McNeish D (2018). Thanks coefficient alpha, we'll take it from here. Psychol Methods.

[51] Hayes AF, Coutts JJ (2020). Use omega rather than Cronbach’s alpha for estimating reliability. But…. Commun Methods Meas.

[52] Tabachnick BG, Fidell LS (2007). Using Multivariate Statistics. 5th edition.

[53] Bland JM, Altman DG (1994). Matching. BMJ.

[54] Sarstedt M, Ringle CM, Hair JF, Homburg C, Klarmann M, Vomberg A (2022). Partial least squares structural equation modeling. Handbook of Market Research.

[55] Cohen J (1992). A power primer. Psychol Bull.

[56] Hair Jr JF, Hult GTM, Ringle CM, Sarstedt M, Danks NP, Ray S (2021). An introduction to structural equation modeling. Partial Least Squares Structural Equation Modeling (PLS-SEM) Using R.

[57] Fornell C, Larcker DF (2018). Evaluating structural equation models with unobservable variables and measurement error. J Mark Res.

[58] Taylor R (2016). Interpretation of the correlation coefficient: A basic review. J Diagn Med Sonogr.

[59] Fletcher TD (2012). QuantPsyc: Quantitative psychology tools, version 1.5. The Comprehensive R Archive Network.

[60] Miller DJ, Vachon DD, Lynam DR (2009). Neuroticism, negative affect, and negative affect instability: Establishing convergent and discriminant validity using ecological momentary assessment. Pers Individ Dif.

[61] Lehne M, Koelsch S (2015). Toward a general psychological model of tension and suspense. Front Psychol.

[62] Dick W, Hagerty N (1971). Topics in Measurement: Reliability and Validity.

[63] Nahm AY, Rao SS, Solis-Galvan LE, Ragu-Nathan TS (2002). The Q-sort method: Assessing reliability and construct validity of questionnaire items at a pre-testing stage. J Mod Appl Stat Methods.

[64] Polit DF (2015). Assessing measurement in health: Beyond reliability and validity. Int J Nurs Stud.

[65] Taherdoost H (2016). Validity and reliability of the research instrument; how to test the validation of a questionnaire/survey in a research. SSRN J.

[66] Eppmann R, Bekk M, Klein K (2018). Gameful experience in gamification: construction and validation of a gameful experience scale [GAMEX]. J Interact Market.

[67] Sabet S, Griwodz C, Möller S (2019). Influence of primacy, recency and peak effects on the game experience questionnaire. Proceedings of the 11th ACM Workshop on Immersive Mixed and Virtual Environment Systems.

[68] Engl S, Nacke LE (2013). Contextual influences on mobile player experience – A game user experience model. Entertain Comput.

[69] Quax P, Beznosyk A, Vanmontfort W, Marx R, Lamotte W (2013). An evaluation of the impact of game genre on user experience in cloud gaming. Proceedings of the 2013 IEEE International Games Innovation Conference.

[70] Sweetser P, Johnson D, Wyeth P, Anwar A, Meng Y, Ozdowska A (2017). GameFlow in different game genres and platforms. Comput Entertain.

[71] Gajadhar B, de Kort Y, IJsselsteijn W (2008). Influence of social setting on player experience of digital games. Proceedings of the CHI Conference on Human Factors in Computing Systems.

[72] Bowman ND, Oliver MB, Rogers R, Sherrick B, Woolley J, Chung MY (2016). In control or in their shoes? How character attachment differentially influences video game enjoyment and appreciation. J Gaming Virtual Worlds.

[73] Melchers KG, Basch JM (2021). Fair play? Sex‐, age‐, and job‐related correlates of performance in a computer‐based simulation game. Int J Sel Assess.

[74] Chappetta KC, Barth JM (2020). Gaming roles versus gender roles in online gameplay. Inf Commun Soc.

