# Supplementary figures and images for: Reliability and Validity of the Arabic Version of the Game Experience Questionnaire: Pilot Questionnaire Study
Source: JMIR Form Res. 2023 Mar 20;7:e42584. doi: 10.2196/42584 (PMC10131659; doi:10.2196/42584)

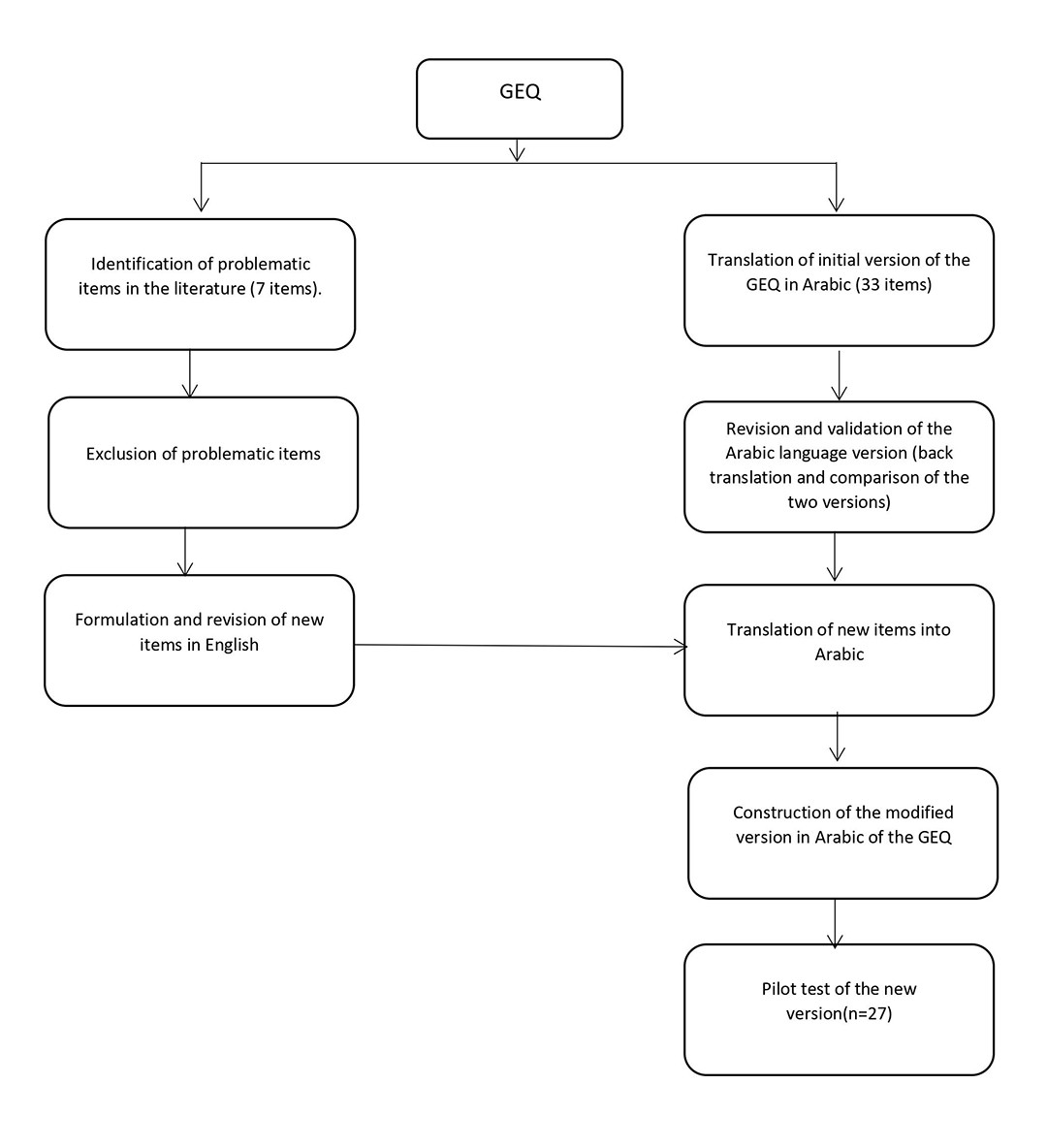

Supplement: Multimedia Appendix 1 [file formative_v7i1e42584_app1.png]

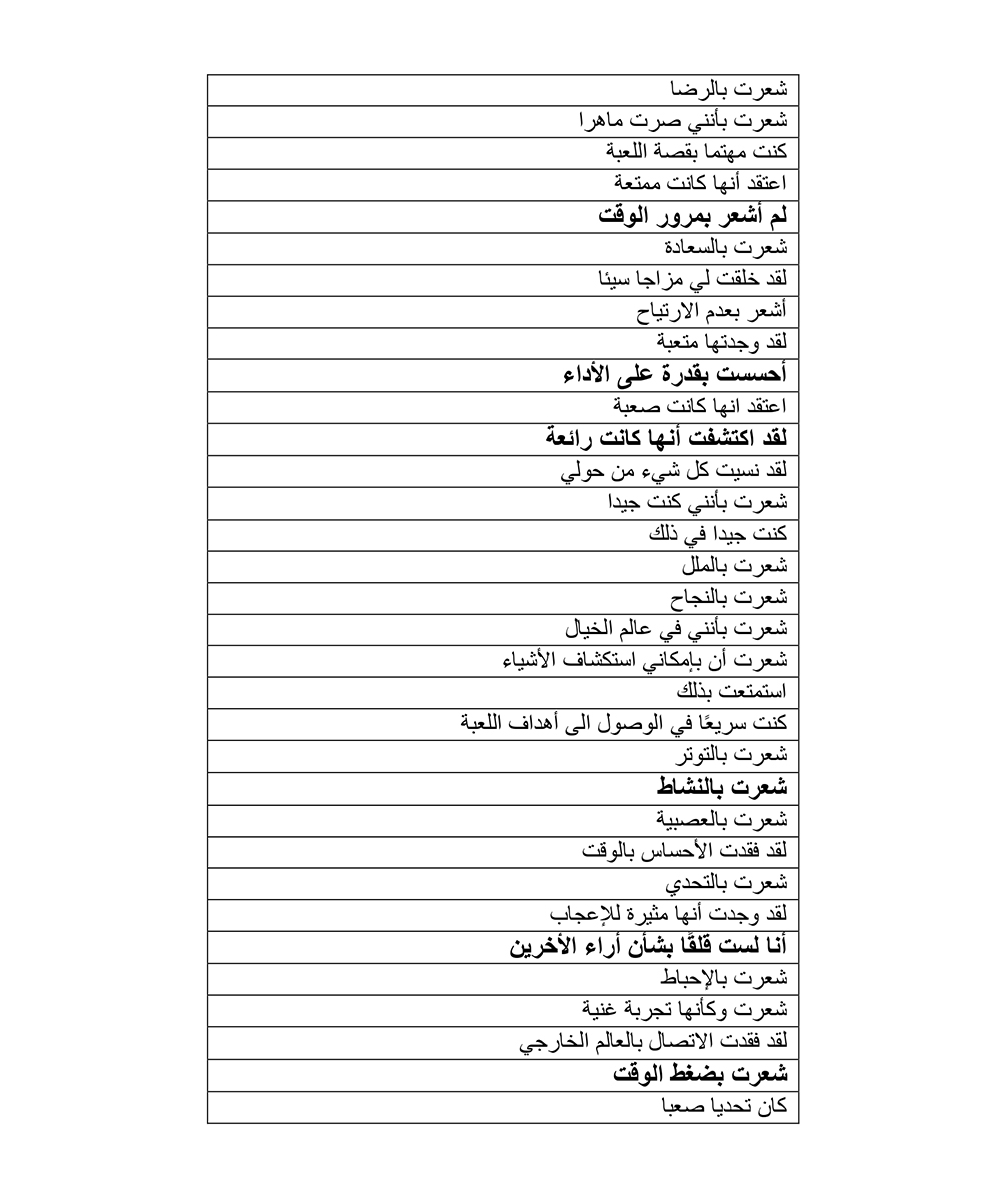

Supplement: Multimedia Appendix 3 [file formative_v7i1e42584_app3.png]
