# Supplementary material for: Reliability and Validity of the Arabic Version of the Game Experience Questionnaire: Pilot Questionnaire Study
Source: JMIR Form Res. 2023 Mar 20;7:e42584. doi: 10.2196/42584 (PMC10131659; doi:10.2196/42584)
Supplement: Multimedia Appendix 2 [file formative_v7i1e42584_app2.pdf]

Original Items	Dimensions	Modified items
**10. I felt competent**	Competence	I felt habile.
**12. It was aesthetically pleasing**	Immersion	I found it fantastic.
**05. I was fully occupied with the game**	Flow	I don't see the time passing
**28. I was deeply concentrated in the game**	Flow	I'm not worried about other people's opinions.
**23. I felt pressured**	Tension	I feel energized.
**33 I had to put a lot of effort into it**	Challenge	It's hard to win.
**08 I thought about other things**	Negative Affect	I feel uncomfortable.

Multimedia Appendix 2. Modified items of the GEQ.
