# Supplementary material for: Reliability and Validity of the Arabic Version of the Game Experience Questionnaire: Pilot Questionnaire Study
Source: JMIR Form Res. 2023 Mar 20;7:e42584. doi: 10.2196/42584 (PMC10131659; doi:10.2196/42584)
Supplement: Multimedia Appendix 4 [file formative_v7i1e42584_app4.pdf]

Gender		Flow		Competence		Positive affect		Negative affect		Immersion		Tension		Challenge	
		M	Sd.	M	Sd	M	Sd.	M	Sd	M	Sd	M	Sd	M	Sd
F	Action (n=99)	2.91	0.77	2.98	0.66	2.85	0.76	1.98	0.89	2.70	0.91	2.18	0.84	2.82	0.82
	Aventure (n=62)	2.98	0.72	2.97	0.67	2.82	0.76	2.09	0.82	2.40	0.97	2.20	0.81	2.75	0.79
	Multiplayers (n=52)	2.78	0.85	2.87	0.76	2.79	0.70	2.02	0.91	2.48	0.82	1.95	0.85	2.71	0.84
	Strategy (n=56)	2.58	0.97	2.88	0.71	2.74	0.69	2.17	0.72	2.57	0.89	2.34	0.71	2.85	0.73
	Others (n=17)	2.35	1.03	2.81	1.05	2.52	1.19	1.95	0.76	2.61	0.91	2.10	0.95	2.77	0.73
M	Action (n=196)	3.02	0.66	3.01	0.69	2.96	0.68	2.02	0.85	2.82	0.78	2.05	0.90	2.86	0.74
	Aventure (n=128)	3.03	0.61	3.01	0.63	2.81	0.79	1.96	0.90	2.83	0.77	1.92	0.98	2.67	0.87
	Multiplayers (n=76)	2.81	0.78	2.85	0.63	2.77	0.73	2.02	0.84	2.40	0.99	2.20	0.76	2.71	0.82
	Strategy (n=67)	2.92	0.83	2.75	0.77	2.85	0.84	1.94	0.80	2.62	0.87	1.96	0.89	2.74	0.71
	Others (n=18)	2.62	1.16	2.68	0.68	2.94	0.71	2.06	0.84	2.42	1.01	2.46	0.80	2.64	0.92

Multimedia Appendix 4. Descriptive statistics for Squares for the Arabic GEQ Sub-scales by game type and Gender.
