# Supplementary material for: Reliability and Validity of the Arabic Version of the Game Experience Questionnaire: Pilot Questionnaire Study
Source: JMIR Form Res. 2023 Mar 20;7:e42584. doi: 10.2196/42584 (PMC10131659; doi:10.2196/42584)
Supplement: Multimedia Appendix 5 [file formative_v7i1e42584_app5.pdf]

| | $\chi 2$ | df | SRMR | RMSEA | CFI |
|---|---|---|---|---|---|
| **GEQ-R** | 504.27 | 265 | 0.068 | 0.053 | 0.895 |
| **GEQ-R with covariances** | 378.21 | 261 | 0.062 | 0.037 | 0.948 |
| **Arabic GEQ** | 454.33 | 1.1 | 0.031 | 0.016 | 0.995 |

Multimedia Appendix 5. Comparison fit index of the Arabic GEQ and the original version.
